# Comparison of Shear Bond Strength and Failure Modes of Transbond XT and Orthomite LC After Thermal Cycling: An In Vitro Study

**DOI:** 10.3390/dj14040239

**Published:** 2026-04-15

**Authors:** Tomoyo Okazaki, Hirohide Kurashina, Yoshinori Ishida, Hitoshi Kawanabe, Kazunori Fukui

**Affiliations:** 1 Department of Orthodontics and Dentofacial Orthopedics, Department of Oral Growth and Development, Ohu University School of Dentistry, Koriyama 963-8611, Fukushima, Japan; 2 Division of Dental Engineering, Department of Biomaterial Science, Ohu University School of Dentistry, Koriyama 963-8611, Fukushima, Japan

**Keywords:** bonding, orthodontic, shear bond strength, thermal cycling, resin cement, failure mode

## Abstract

**Background**/**Objectives**: Bond reliability is essential in orthodontic treatment, as temperature fluctuations in the oral environment can weaken adhesive interfaces and increase the risk of bracket failure. However, direct comparison of the long-term durability of commonly used orthodontic resin cements under thermocycling conditions is limited. Therefore, the present study aimed to evaluate and compare the shear bond strength (SBS) and failure modes of Transbond™ XT and Orthomite™ LC before and after thermal cycling (Tc). **Methods**: A total of 60 bovine enamel specimens were used in this study. Specimens were bonded with either Transbond XT or Orthomite LC under standardized conditions. SBS was measured at 24 h (Tc0) and after 5000 thermal cycles (Tc5000). Failure modes were classified as adhesive (A), enamel cohesive (B), or bracket cohesive (C) failure. Statistical analyses included the Mann–Whitney U test for SBS and Fisher’s exact test for failure mode distribution. **Results**: At Tc0, there was no significant difference in SBS between the two cements (*p* > 0.05). After Tc5000, Orthomite LC showed significantly higher SBS than Transbond XT (*p* = 0.00368). Failure mode analysis revealed that, after Tc, Transbond XT exhibited a higher incidence of adhesive failures (A), whereas Orthomite LC predominantly demonstrated bracket cohesive failures (C) (*p* = 0.00020). **Conclusions**: Orthomite LC demonstrated greater resistance to thermal cycling–induced bond degradation compared with Transbond XT, likely due to differences in resin monomer composition and interface stability.

## 1. Introduction

In orthodontic treatment, esthetics is always an important consideration. Even for metal brackets, efforts have been made to improve appearance by reducing the size of stainless-steel brackets and minimizing the visible metallic components [1]. To meet increasing aesthetic demands, ceramic brackets were introduced in the mid-1980s [2]. Currently, ceramic brackets are primarily fabricated from aluminum oxide (Al_2_O_3_) and are available in two types: monocrystalline and polycrystalline. Compared with metal brackets, ceramic brackets exhibit distinctive characteristics, including excellent biocompatibility, superior esthetics, resistance to chemical and thermal changes, and sufficient bond strength [3,4]. More recently, zirconia-based ceramic brackets have been introduced in orthodontics to improve mechanical strength and esthetics, reflecting the growing diversity of ceramic bracket materials. Recent studies comparing the shear bond strength (SBS) of metal and ceramic orthodontic brackets have demonstrated that ceramic brackets can achieve clinically acceptable, or even higher, SBS values depending on bonding protocols [5], emphasizing the need for reliable adhesive systems for ceramic brackets. In orthodontic treatment, brackets must be bonded to the tooth surface using various cements. In recent years, resin-based cements have been primarily used, owing to their ease of handling and high bond strength [6]. As orthodontic treatment often extends over several years, the adhesive bond must withstand mechanical forces, such as orthodontic and masticatory loads, and fluctuations in oral temperature and humidity, without debonding [7,8]. Moreover, as brackets are bonded to all teeth in many cases, the tooth surface preparation process can be time-consuming, and complex multi-step procedures may increase the risk of bonding failure [9]. Consequently, the loss of bond strength or bracket detachment remains a major concern in orthodontics [10]. To address these issues, new resin-based cements that do not require the application of a separate primer after acid etching of the enamel surface have been developed [11]. Among these, Transbond™ XT (3M Unitek, Monrovia, CA, USA) and Orthomite™ LC (Sun Medical, Moriyama, Shiga, Japan) are designed to bond after enamel acid etching alone, according to the manufacturers’ instructions. However, research evaluating their bond strength to enamel or comparing their performance with conventional bonding protocols is scarce.

Bond stability in orthodontics is a critical factor directly related to treatment efficiency and prognosis. Insufficient bond strength can lead to bracket failure during treatment, necessitating rebonding and prolonging the treatment period. Conversely, excessive bond strength may increase the risk of enamel damage during debonding; therefore, an optimal range of bond strength is required. Resin infiltration into etched enamel is essential for micromechanical retention, which directly affects the bond strength of orthodontic brackets. The ability of adhesive materials to flow into enamel microporosities depends on their viscosity and flow properties. Recent evidence has demonstrated that resin infiltration significantly enhances SBS and contributes to the preservation of enamel integrity during orthodontic treatment [12].

Conventional Transbond XT is a three-step bonding system involving acid etching, followed by application of a dedicated primer, and can achieve high initial bond strength. However, thermal cycling and long-term water storage can cause degradation at the adhesive interface, reducing retention. In contrast, LC Orthomite is a one-step, primerless bonding system containing hydrophilic monomers (e.g., HEMA) within the paste, allowing direct application after acid etching. The inclusion of multiple low-viscosity resin monomers is expected to enhance penetration into the micro-etched enamel surface and improve chemical bonding with enamel components, potentially resulting in superior long-term durability. Nevertheless, few studies have directly compared the initial bond strength and long-term durability of these two bonding systems after thermal cycling.

Therefore, the present study aimed to measure and statistically compare the SBS of Transbond XT and LC Orthomite after 24 h water storage and after 5000 thermal cycles. The null hypothesis was that no significant differences would be observed in SBS or failure modes between the two adhesive systems before or after thermal cycling.

## 2. Materials and Methods

All experimental procedures were conducted under standardized conditions to support reproducibility. The study design, sample allocation, bonding procedures, storage conditions, and testing protocols are described in detail below. Two types of light-cured orthodontic resin cements were used for bracket bonding: Transbond™ XT (3M Unitek, Monrovia, CA, USA) and Orthomite™ LC (Sun Medical, Moriyama, Shiga, Japan). For enamel surface etching, Transbond XT was conditioned with Transbond XT Etching Gel (Solventum, Monrovia, CA, USA; Product No. 11030525; 65% phosphoric acid), and Orthomite LC was conditioned with Orthomite LC Etching Gel (Sun Medical, Moriyama, Japan; Product No. FX12F; 37% phosphoric acid). The adhesives used were Transbond XT Adhesive Primer (3M Unitek) and Orthomite LC Paste (Sun Medical, Moriyama, Japan). The composition of each material is shown in Table 1.

All specimens were randomly assigned to each experimental group to minimize selection bias. The brackets used were Clarity™ ADVANCED Ceramic Brackets for maxillary central incisors, 0.022-inch straight-edgewise type (3M Unitek, Monrovia, CA, USA). Polymerization of the adhesive resin was performed using a PEN Bright LED curing unit (Shofu, Kyoto, Japan).

Mandibular first incisors from cattle were obtained from a commercial meat-processing facility. All teeth were collected as by-products of the food industry; therefore, no animals were sacrificed specifically for this study, and ethical approval was not required according to institutional guidelines. Teeth were used within 6 months of slaughter and were collected from animals less than 30 months of age. The roots were sectioned, and the crowns were embedded in acrylic resin with the labial surface widely exposed. The enamel surfaces were sequentially polished under water cooling with 320- and 600-grit silicon carbide papers to obtain a flat, uniformly polished enamel surface. Enamel-etching protocols and application procedures were performed strictly according to the manufacturers’ instructions, following standard clinical bonding practice. Sixty specimens were used in this study and were randomly allocated to four experimental groups (*n* = 15 per group) according to adhesive system (Transbond XT or Orthomite LC) and storage condition (0 thermocycles [Tc0] or 5000 thermocycles [Tc5000]). The thermocycling protocol (5000 cycles between 5 °C and 50 °C) was selected as a commonly used in vitro aging method to simulate thermal stresses encountered in the oral environment.

Before applying each bonding system, the enamel surface was cleaned, dried, and subjected to the designated surface treatment. In the Transbond XT groups, the enamel surface was etched with 65% phosphoric acid for 15 s, rinsed with water for 30 s, dried, and then coated with Transbond XT primer. In the Orthomite LC group, the enamel surface was etched with 37% phosphoric acid for 30 s, rinsed with water for 30 s, and then dried.

**Figure 1:**
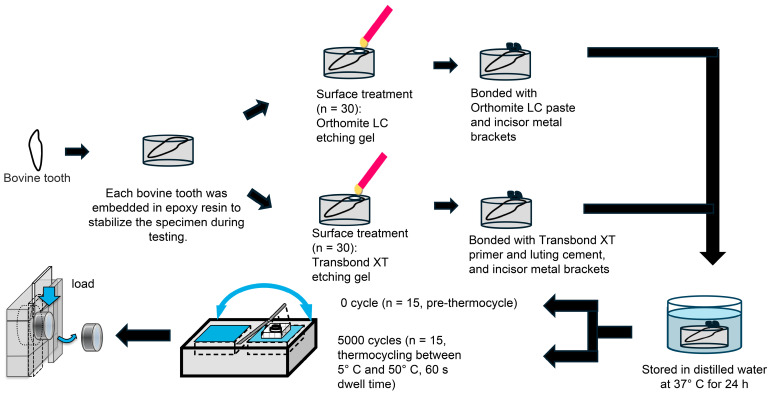
Experimental procedure for bonding maxillary central incisor brackets to bovine enamel. Specimens were treated with either Orthomite LC or Transbond XT systems, then tested after 24 h of water storage at 37 °C (T0 cycle) or after 5000 thermal cycles between 5 °C and 50 °C.

As Orthomite LC contains a primer within the resin cement, no separate primer was applied to the enamel surface. In all groups, the adhesive resin cement was applied to the bracket base and pressed onto the prepared enamel surface. After removal of excess resin, polymerization was performed from the mesial, distal, occlusal, and gingival directions for 10 s each (total curing time, 40 s) using the same PEN Bright LED curing unit (Shofu, Kyoto, Japan; wavelength range: 440–490 nm). All bonding procedures were performed by a single operator under standardized conditions to reduce procedural variability. After bonding, specimens assigned to the Tc0 groups were immersed in distilled water at 37 °C for 24 h. SBS was measured immediately following this 24 h storage period to serve as the baseline condition (i.e., no thermocycling). Specimens in the Tc5000 groups were subjected to 5000 thermal cycles between 5 °C and 55 °C, with a 60 s dwell time in each bath. SBS was measured using a universal testing machine (Type 5566, Instron, Canton, MA, USA) at a crosshead speed of 0.5 mm/min (Figure 1).

Failure mode classification: Post-debonding, failure modes were classified according to fracture location using a modified system derived from previously reported adhesive failure analyses. Failure modes were categorized as follows: (A) adhesive failure, defined as separation at the enamel-adhesive resin interface; (B) mixed failure, defined as adhesive failure combined with cohesive fracture within the adhesive or enamel; and (C) cohesive failure, defined as fracture occurring primarily within the adhesive resin or the ceramic bracket. This classification differs from the conventional adhesive remnant index in that it emphasizes fracture location rather than the amount of residual adhesive on the enamel surface. All specimens were evaluated by a single calibrated examiner using a digital microscope at 40× magnification to maintain consistency. Statistical analyses were performed using SPSS software (version 26.0; IBM Corp., Armonk, NY, USA). Data distribution was assessed for normality using the Shapiro–Wilk test. As the SBS data did not follow a normal distribution, nonparametric statistical methods were applied.

SBS between adhesive systems under each storage condition (Tc0 and Tc5000) was compared using the Mann–Whitney U test. Failure mode distributions were assessed using Fisher’s exact test. Statistical significance was set at *p* < 0.05.

## 3. Results

**Figure 2:**
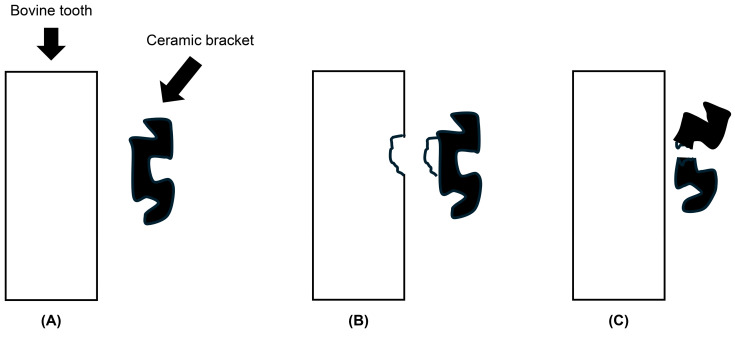
Classification of bond failure modes. (**A**) Separation at the interface between the bovine tooth and the luting agent. (**B**) Cohesive failure within the bovine tooth, accompanied by interface separation. (**C**) Cohesive failure within the ceramic bracket.

The median SBS values for each group are summarized in Table 2. At Tc0, there was no statistically significant difference in SBS between Transbond™ XT (11.9 MPa, IQR 3.85) and Orthomite™ LC (11.2 MPa, IQR 12.15) (Mann–Whitney U test, *p* > 0.05). After 5000 thermal cycles (Tc5000), the SBS of Transbond XT decreased markedly to 5.8 MPa (IQR 2.2), whereas Orthomite LC maintained a higher value of 10.0 MPa (IQR 4.25). This difference was statistically significant (Mann–Whitney U test, *p* = 0.00368), indicating that Orthomite LC exhibited enhanced resistance to thermocycling-induced degradation. Failure mode distributions are presented in Figure 2 and Table 3. At Tc0, Transbond™ XT exhibited 2 adhesive failures (A), 7 mixed failures (B), and 6 cohesive failures (C), whereas Orthomite™ LC showed 1/6/8 failures (A/B/C). No significant difference in failure mode distribution was observed between groups (Fisher’s exact test, *p* = 1.000).

At Tc5000, Transbond XT predominantly exhibited adhesive failures (10/3/2, A/B/C), whereas Orthomite LC demonstrated no adhesive failures and demonstrated 0/5/10 failures (A/B/C). This difference was statistically significant (Fisher’s exact test, *p* = 0.00020). Each specimen was assigned exclusively to either the Tc0 or the Tc5000 condition; therefore, no specimen underwent both tests. These results indicate that Orthomite LC maintains superior interfacial stability after thermal cycling compared with Transbond XT.

## 4. Discussion

The maxillary central incisor is considered the most ideal tooth for orthodontic bonding experiments, as its labial surface is nearly flat, eliminating the need to account for curvature effects during bracket bonding. However, in recent years, growing interest in esthetics and advances in treatment techniques have made it increasingly difficult to obtain human maxillary central incisors for research purposes [13]. All mammalian teeth share similar histological structures. According to Nakamichi et al., although bovine enamel tends to exhibit slightly lower bond strength than human enamel, this difference is not statistically significant. Therefore, in this study, bovine enamel was used as a substitute for human enamel [14,15].

To reduce the time required for orthodontic bracket bonding procedures, resin cements that do not require a separate adhesive application have been introduced. These cements exhibit SBSs comparable to or slightly lower than those of conventional multi-step bonding systems [16]. The minimum clinically acceptable SBS required to maintain orthodontic brackets in the oral cavity was reported to be 5.9–7.8 MPa [7]. In the present study, no statistically significant differences in SBS were observed among the experimental groups; however, all groups met this proposed threshold, regardless of surface treatment method.

The bond strength between the adhesive and the enamel surface is influenced by the intrinsic strength of the resin and its ability to penetrate the microporosities of the enamel surface. In addition, the flowability of the resin cement may influence its ability to infiltrate these microstructures, with higher flowability facilitating deeper penetration into etched enamel and potentially enhancing micromechanical retention and bond strength. This finding is supported by recent studies demonstrating that flowable composite resins exhibit favorable SBS compared with conventional materials [17,18]. However, this factor was not directly evaluated in the present study and should be investigated in future research. Failure mode analysis was performed using optical microscopy at 40× magnification, which may limit the detection of microscopic interfacial defects or detailed fracture patterns. However, this method is sufficient for distinguishing major failure modes such as adhesive, cohesive, and mixed failures and is consistent with previous in vitro studies employing stereomicroscopy at similar magnifications [19,20]. The smooth enamel surface becomes roughened by acid etching, creating irregular micro-etch patterns that increase the available bonding surface area [21,22]. The surface tension of etched enamel is approximately 72 mN/m, approximately twice that of the unetched state, enabling hydrophobic resin solutions to infiltrate the micro-pores via capillary action. These characteristics likely explain why all experimental groups in the present study demonstrated high SBS, regardless of surface treatment [23].

The Mann–Whitney U test revealed no significant difference in SBS between the Transbond XT and LC Orthomite groups under the 24 h (Tc0) condition. In contrast, under the Tc5000 condition, the LC Orthomite group exhibited significantly higher bond strength than the Transbond XT group (*p* = 0.00368), suggesting that LC Orthomite maintained strong adhesion even after thermocycling. This result further suggests greater resistance of LC Orthomite to thermocycling-induced degradation. Nonetheless, as resin composition and interfacial characteristics were not directly assessed in this study, the mechanisms underlying this behavior cannot be definitively determined. Failure mode analysis (Table 3) revealed that under the Tc0 condition, both groups predominantly exhibited type B (cohesive failure within the tooth structure) and type C (cohesive failure within the bracket), with only a few cases of type A (adhesive failure). Fisher’s exact test revealed no significant difference in distribution between the groups (*p* = 1.000). However, under the Tc5000 condition, the Transbond XT group exhibited a marked increase in type A failures (10/15), with decreases in types B and C. In contrast, the LC Orthomite group exhibited no type A failures, with type C failures being the most frequent (10/15). Fisher’s exact test indicated a significant difference in failure mode distribution between the groups under Tc5000 (*p* = 0.00020). These findings indicate that thermocycling altered failure mode distribution differently between the two adhesive systems. However, detailed characterization of interfacial adhesion was beyond the scope of this study. Collectively, these results indicate that Transbond XT was more susceptible to thermocycling-induced degradation than LC Orthomite, whereas LC Orthomite demonstrated greater retention of SBS. Previous studies have suggested that differences among adhesive systems, including material composition, may influence bond durability and aging behavior. Nevertheless, as the present study did not directly evaluate resin monomer composition or interfacial chemistry, any interpretation regarding the role of material composition should be considered speculative and based on existing literature rather than direct evidence from this study [8]. Instead, the observed differences are likely related to variations in the material composition of each product. According to the manufacturers, Transbond XT paste contains 70–80 wt% filler and 15–30 wt% resin monomers, whereas the Transbond XT primer contains the hydrophobic monomer Bis-GMA and the diluent TEGDMA, which helps compensate for the relatively low monomer content of the paste, enhancing adhesion to the tooth surface.

Hosein et al. reported that the higher the bond strength of orthodontic cements, the greater the amount of residual resin left after bracket removal [24,25]. In the present study, all groups exhibited predominantly mixed failure modes. From a clinical perspective, comparing the residual cement area on the tooth surface after debonding may help in selecting a cement that facilitates smoother enamel surfaces after bracket removal. Previous research has also assessed the reliability of qualitative and quantitative assessments of adhesive remnants after bracket debonding, highlighting the importance of accurate evaluation of residual adhesive on enamel surfaces [26]. Moreover, the sustained SBS of Orthomite LC after thermal cycling may be attributed to its specific monomer composition and filler loading, which potentially enhance the stability of the adhesive interface. The inclusion of primer components within the resin cement likely contributes to a more robust chemical bond, thereby reducing susceptibility to hydrolytic degradation. This structural integrity is further reflected in the failure mode analysis, in which a higher incidence of bracket cohesive failure was observed than with Transbond XT.

This study has certain limitations. First, bovine teeth were used instead of human enamel, and although previous reports indicate comparable bonding behavior, slight structural differences may influence absolute bond strength values. Second, only two adhesive systems and one type of ceramic bracket were evaluated; therefore, the results may not be generalizable to other orthodontic materials. Third, the chemical composition and interfacial bonding mechanisms of the adhesives were not directly analyzed; therefore, interpretations regarding material performance should be made with caution. Fourth, surface morphology and adhesive interfaces were not evaluated using advanced imaging techniques such as scanning electron microscopy, which could provide further insight into bonding mechanisms. Fifth, environmental factors such as saliva, pH fluctuations, and bacterial biofilms were not simulated, although these factors are known to influence adhesive degradation in vivo. However, this study was designed as a controlled in vitro experiment to isolate the effects of the tested materials under standardized conditions, thereby minimizing confounding variables. Future studies incorporating artificial saliva, pH cycling, and biofilm models are warranted to better reproduce the clinical environment and evaluate long-term performance. Sixth, as this was an in vitro study, the experimental conditions do not fully replicate the complex biological and mechanical environments of the oral cavity. Seventh, mechanical fatigue or cyclic loading was not evaluated, although orthodontic brackets are subjected to repeated masticatory and orthodontic forces in the oral cavity. Eighth, the limited sample size of 15 specimens per group may limit the statistical power to detect subtle differences between adhesive systems. Finally, the aging protocol included only 5000 thermal cycles, which may not fully replicate long-term intraoral conditions. Therefore, future studies should include a wider variety of adhesive systems and bracket materials and, when possible, human enamel substrates to improve clinical relevance. Moreover, more comprehensive aging protocols, including extended thermocycling, long-term water storage, and mechanical fatigue testing, should be implemented to more accurately simulate oral environments. Interfacial analyses using techniques such as scanning electron microscopy may also provide further insight into the mechanisms responsible for thermal degradation and material performance.

## 5. Conclusions

This study demonstrated that both bonding systems demonstrated adequate SBS after 24 h; however, LC Orthomite exhibited significantly greater resistance to thermocycling-induced degradation than Transbond XT. The combination of higher post-cycling bond strength and favorable failure mode distributions suggests that LC Orthomite may provide superior long-term adhesive stability in orthodontic applications.

## Figures and Tables

**Table 1:** Assessment of the materials tested in this study.

Material/Trade Name	Manufacturer	Lot.	Composition (%)
Etchant			
Trans bond XT Etching gel	Solventum, Monrovia, CA, USA	11030525	Phosphoric acid, 65%
Orthomite LC Etching gel	Sun Medical, Moriyama, Japan	FX12F	Phosphoric acid, 35–45%
Primer			
Transbond XT primer	Solventum, Monrovia, CA, USA	10826863	Bis-GMA and TEGDMA
Luting material			
Transbond XT paste	Solventum, Monrovia, CA, USA	10952015	Silane-treated quartz Bis-GMA Dichlorodimethylsilane Silane-treated silica Diphenyliodonium hexafluorophosphate
Orthomite LC Paste	Sun Medical, Moriyama, Japan	FK2728	2-Hydroxyethyl methacrylate 2-Propenoic acid, 2-methyl-, 7,7,9-trimethyl-4,13-dioxo-3,14-dioxa-5,12-diazahexadecane-1,16-diyl ester 2-Propenoic acid, 2-methyl-, 1,3-phenylenebis

**Table 2:** Shear bond strength between bovine teeth and the luting agent cements (in MPa).

	Tc0		Tc5000		M-W	Post-/Pre-Ratio (%)
	Median	IQR	Median	IQR		
Trans bond XT	11.9	3.85	5.8	2.2	NS	48.7
Orthomite LC	11.2	12.15	10.0	4.25	S	89.3

Values are shown as median (IQR). M-W: Mann–Whitney U test; NS: not significant (*p* > 0.05); S: significant (*p* < 0.05). Post-/Pre-ratio indicates the percentage of SBS retained after 5000 thermal cycles.

**Table 3:** Failure modes of specimens.

	Tc0			Tc5000			*p*-Value (Fisher’s Exact Test)
	A	B	C	A	B	C	
Trans bond XT	2	7	6	10	3	2	Tc0: 0.77
Orthomite LC	1	6	8	0	5	10	Tc5000: <0.001

Distribution of failure modes classified as adhesive (A), mixed (B), and cohesive (C) for each adhesive system at Tc0 and Tc5000.

## Data Availability

The original contributions presented in this study are included in the article. Further inquiries can be directed to the corresponding author.

## Abbreviations

SBS	Shear Bond Strength
Tc	Thermal Cycling
Tc0	Baseline Condition with 0 Thermal Cycles
Tc5000	Condition After 5000 Thermal Cycles
MPa	Megapascal
IQR	Interquartile Range
SEM	Scanning Electron Microscopy (mentioned for future studies)
